# Mercury, selenium and fish oils in marine food webs and implications for human health

**DOI:** 10.1017/S0025315415001356

**Published:** 2015-09-08

**Authors:** Matthew O. Gribble, Roxanne Karimi, Beth J. Feingold, Jennifer F. Nyland, Todd M. O'Hara, Michail I. Gladyshev, Celia Y. Chen

**Affiliations:** 1 Department of Preventive Medicine, University of Southern California Keck School of Medicine, Los Angeles, CA, USA; 2 School of Marine and Atmospheric Sciences, Stony Brook University, Stony Brook, NY, USA; 3 Department of Environmental Health Sciences, University at Albany School of Public Health, State University of New York, Rensselaer, NY, USA; 4 Department of Pathology, Microbiology and Immunology, University of South Carolina School of Medicine, Columbia, SC, USA; 5 Department of Veterinary Medicine, College of Natural Science and Mathematics, University of Alaska Fairbanks, Fairbanks, AK, USA; 6 Institute of Biophysics of Siberian Branch of Russian Academy of Sciences, Akademgorodok, Krasnoyarsk, Russia; 7 Siberian Federal University, Krasnoyarsk, Russia; 8 Department of Biological Sciences – Dartmouth College, Hanover, NH, USA

**Keywords:** Oceans and human health, OHH, mercury, selenium, fish oils, n−3 fatty acids, eicosapentaenoic acid, docosahexaenoic acid, ecotoxicology, public health

## Abstract

Humans who eat fish are exposed to mixtures of healthful nutrients and harmful contaminants that are influenced by environmental and ecological factors. Marine fisheries are composed of a multitude of species with varying life histories, and harvested in oceans, coastal waters and estuaries where environmental and ecological conditions determine fish exposure to both nutrients and contaminants. Many of these nutrients and contaminants are thought to influence similar health outcomes (i.e., neurological, cardiovascular, immunological systems). Therefore, our understanding of the risks and benefits of consuming seafood require balanced assessments of contaminants and nutrients found in fish and shellfish. In this paper, we review some of the reported benefits of fish consumption with a focus on the potential hazards of mercury exposure, and compare the environmental variability of fish oils, selenium and mercury in fish. A major scientific gap identified is that fish tissue concentrations are rarely measured for both contaminants and nutrients across a range of species and geographic regions. Interpreting the implications of seafood for human health will require a better understanding of these multiple exposures, particularly as environmental conditions in the oceans change.

The world's oceans support marine fisheries for commercial, recreational and subsistence uses, and thus are directly linked to human health through fish consumption (i.e. Bergé & Barnathan, 2005; Kite-Powell *et al*., 2008; Halpern *et al*., 2012; Moore *et al*., 2013; Tacon & Metian, 2013). Fish comprise an important source of animal protein for much of the world's human population, and in the next decade, total production from wild fisheries and aquaculture is expected to exceed production of beef, pork or poultry (FAO/WHO, 2011). In 2010, fish accounted for 16.7% of the world's intake of animal protein, and the world fish food supply grew 3.2% per year from 1961–2012, nearly doubling from an average of 9.9 kg per capita to 19.2 kg per capita (FAO, 2014, pp. 3–4).

The ability of the global population to obtain healthful marine-derived food is dependent on well-managed ecosystems. A broad interdisciplinary approach is needed to understand the connections between the marine environment and human health (Kite-Powell *et al*., 2008; Moore *et al*., 2013), particularly for evaluating the risks and benefits of consuming seafood. This necessarily requires expertise from marine science as well as public health and biomedical science. This paper is authored by an interdisciplinary group comprising marine and human health scientists who have shared their expertise to synthesize current knowledge on the benefits and risks of consuming marine organisms as routes of human exposure to combinations of fish oils, selenium and the global contaminant mercury, particularly its highly bioavailable and toxic form, methylmercury. Other potential compounds of interest in marine organisms, including organohalogens, natural toxins, arsenicals, trace essential elements and vitamins are beyond the scope of this review; however, introductions to such topics are available elsewhere (Jeandel & Minster, 1987; Edmonds & Francesconi, 1993; Neff, 1997; Garthwaite, 2000; Lail *et al*., 2007; Guglielmo *et al*., 2009; Shaw & Kannan, 2009; Yogui & Sericano, 2009; Dickey & Plakas, 2010; Buck *et al*., 2011; Cusick & Sayler, 2013; Prego-Faraldo *et al*., 2013; Skjånes *et al*., 2013; Ahrens & Bundschuh, 2014; Alonso *et al*., 2014; Sañudo-Wilhelmy *et al*., 2014). This review provides a limited overview of select dimensions of marine seafood chemical content, and demonstrates the multidisciplinary issues at the interface of Oceans and Human Health (OHH). It does not set out to provide a comprehensive review of seafood content or the overall health implications of seafood consumption. The collaboration of the co-authors of this paper, hailing from diverse disciplinary backgrounds including veterinary medicine, toxicology, immunology, epidemiology, ecology, toxicology and geography, also exemplifies the goals of the OHH initiative which includes the sharing of insights and priorities across research communities (European Marine Board, 2013).

## HEALTH BENEFITS OF FISH CONSUMPTION

Fish and shellfish contain protein, long-chain omega-3 fatty acids, vitamins, minerals and trace elements such as calcium and magnesium (Tacon & Metian, 2013). Seafood has the highest concentration of long-chain omega-3 polyunsaturated fatty acids (PUFAs), including eicosapentaenoic acid (EPA) and docosahexaenoic acid (DHA), of any foods (Tacon & Metian, 2013). EPA and DHA show beneficial associations with cardiovascular phenotypes including blood pressure (Campbell *et al*., 2013), vascular endothelial function (Xin *et al*., 2012), arterial stiffness (Pase *et al*., 2011) and heart rate variability (Xin *et al*., 2013). Fish or fish oil intake is also associated with decreased weight and waist circumference (Bender *et al*., 2014). Possible impacts of EPA and DHA on cholesterol in humans are unclear. Among persons with diabetes, fish oil supplementation may be associated with lower triglycerides and lower levels of very low density lipoprotein (VLDL) cholesterol, but with higher levels of low density lipoprotein (LDL) cholesterol (Hartweg *et al*., 2008). In dialysis patients, there are also associations of fish oil supplements with lower triglycerides, but also higher high density lipoprotein (HDL) cholesterol, and no association with LDL cholesterol (Zhu *et al*., 2014). However, the relationship of EPA and DHA to hard cardiovascular endpoints is less clear. A pooled meta-analysis of 68,680 fish oil supplement clinical trial participants, many of whom (more than half of the trials) had pre-existing cardiovascular disease and were being followed for a second event, did not show evidence for lower risk of mortality (from any cause), cardiac death, myocardial infarction or stroke (Rizos *et al*., 2012). In contrast, many observational studies report a decrease in cardiovascular disease and all-cause mortality with higher fish oil intake (Wang *et al*., 2006). The discrepancy between the clinical trial and the observational study results may reflect differences in study populations, or may suggest that another nutrient in fish (or an interacting cofactor in fish) is responsible for some of the cardiovascular benefits attributed to fish oils.

In addition to their possible relevance for cardiometabolic diseases, EPA and DHA fatty acids also may be associated with many other health outcomes. For example, observational studies suggest a lower risk of breast cancer with higher exposure (Zheng *et al*., 2013). DHA is essential for ophthalmological and neurological development (Uauy *et al*., 2001; Janssen & Kiliaan, 2014) and fish oil supplements may be associated with cognitive development among infants (Jiao *et al*., 2014). Among women who previously had delivered a pre-term baby, fish oil supplements appeared to be associated with longer latency and greater weight at birth of the child but did not appear to be associated with differences in risk of another pre-term birth (Saccone & Berghella, 2015).

Selenium, present in marine biota including fish and mussels (Outzen *et al*., 2015), has biological effects that are dose-dependent: at low doses, selenium is an essential nutrient used in selenoproteins such as glutathione peroxidase (Barceloux, 1999), but at higher doses, selenium might be toxic to a range of animals including humans (Barceloux, 1999; Hoffman, 2002; Lemly, 2002; Adams *et al*., 2003; Ackerman & Eagles-Smith, 2009; Rigby *et al*., 2010; Hladun *et al*., 2013; Thomas & Janz, 2014), although the dose-response of selenium toxicity differs across animal species (Ackerman & Eagles-Smith, 2009). In humans, the health effects of selenium (total selenium and selenium species) are controversial, with ongoing research into possible elevations or decreases in risk of various health outcomes according to selenium intake (Sabino *et al*., 2013). A recent Cochrane review (a comprehensive review in medical sciences that aims to summarize published and unpublished data on a topic) of selenium and cancer prevention found heterogeneous studies furnishing no overall evidence that selenium reduces cancer risk (Vinceti *et al*., 2014).

## HAZARDS OF MERCURY

Although seafood provides important nutritional benefits, there may also be hazards from contaminants such as mercury. Neurological impacts of high methylmercury exposure were described in mass poisoning events in Minamata Bay, Japan (Harada, 1995) from consumption of seafood contaminated by effluent from a chlor-alkali facility. ‘Minamata disease’ was characterized by deficits in sensation, vision, hearing, coordination (ataxia) and other problems associated with neurological function (Eto *et al*., 1999; Uchino *et al*., 2005). Children who had high *in utero* exposures suffered many neurotoxic effects including cerebral palsy, mental retardation, sensorimotor dysfunction and low birth weight (Chapman & Chan, 2000; Karagas *et al*., 2012). At lower doses, the neurological effects of methylmercury are less clear (Axelrad *et al*., 2007; Karagas *et al*., 2012).

### Neurodevelopmental toxicity of mercury

Methylmercury neurotoxicity from consumption of seafood has been the focus of birth cohorts in the Faroe Islands, Seychelles and elsewhere (Table 1). In the Faroe Islands, where much of the mercury was acquired from consumption of marine mammals contaminated by organochlorines, there was an inverse association between mercury in cord blood and children's performance on developmental tests (Grandjean *et al*., 2001, 2014). However, in the Seychelles, where much of the mercury was from fish, overall associations between foetal exposure to mercury and neurodevelopmental impairments were generally not observed (Carocci *et al*., 2014). However, at 9 years of age there appeared to be possible differences in fine motor function at higher levels of mercury exposure (Davidson *et al*., 2006; van Wijngaarden *et al*., 2006; Mergler *et al*., 2007), and evidence for interactions between fatty acids and mercury for cognitive processes (Strain *et al*., 2015). Emerging research suggests that genetic polymorphisms and epigenetic processes may account for some of the inter-individual variations of health effects given exposures (reviewed in Basu *et al*., 2014). A recent systematic review examined the associations between exposure to methylmercury from seafood consumption and developmental neurotoxicity from 164 studies in 43 countries and found that mercury might be impacting the health of Arctic and riverine populations near gold mining sites, and might also be relevant for public health in highly populated coastal regions (Sheehan *et al*., 2014).

**Table 1.** Major cohort studies examining early-life methylmercury (MeHg) and total mercury (Hg) exposure and neurodevelopment in children. IQR, inter-quartile range (25th to 75th percentile).

Population	Study sample	Measure of exposure	Average exposure (ppm)	Neurological associations
Faroe Islands (Grandjean *et al*., 1997; Grandjean *et al*., 2014)	1022 singleton births, 917 children at age 7	Hg concentrations in maternal hair at delivery, cord blood, child blood and hair at age 7 years	Geometric mean and IQR at 7 years: hair Hg 3.03 (1.68–6.33), maternal hair Hg in pregnancy: 4.35 (2.63–42.2)	Neurodevelopmental deficits (i.e. visuospatial memory) at birth and early school years when comparing high and low exposure groups
Italy (Deroma *et al*., 2013)	149 children	Total Hg and MeHg in maternal hair and breast milk and child's hair at 7–9 years	Median maternal hair Hg (total): 1.38	Children with high prenatal Hg exposure had lower verbal, scale and performance IQ than children with low prenatal Hg exposure, but this difference was not significant. In contrast, children's fresh fish consumption was positively associated with scale and performance IQ
Italy (Valent *et al*., 2013)	606 children at 18 months of age	Maternal and child fish intake; total Hg in maternal hair and blood during pregnancy, umbilical cord bood, and breast milk	Mean maternal hair Hg: 1.06	No evidence of prenatal Hg exposure linked to children's neurodevelopment. Children's fish intake, but not maternal PUFAs (EPA, DHA and other fatty acids), were positively associated with neurodevelopmental test scores
United States – Massachusetts (Oken *et al*., 2005)	135 infant-mother pairs	Self reported Fish consumption during 2nd trimester of pregnancy, maternal total Hg in hair at delivery	Mean maternal hair Hg: 0.55 (range 0.02–2.38)	Increased maternal fish intake during pregnancy associated with increased infant cognition at 6 months of age. This association was stronger after adjusting for maternal hair Hg at delivery. Higher Hg levels were associated with lower infant cognition at 6 months of age
Seychelles (Davidson *et al*., 1998; Myers *et al*., 2003; Myers *et al*., 2009)	Seychelles Child Development Study Main Cohort: 770 mother-child pairs (children through 107 months)	MeHg exposure (measured as total Hg in hair) from maternal hair, and children's hair at 66 and 107 months	Mean maternal hair Hg: 6.8 Mean child hair-Hg at 66 months: 6.5 (sd: 3.3); at 107 month: 6.1 (sd: 3.6)	Hg not consistently associated with neurodevelopmental outcomes
Seychelles (Strain *et al*., 2015)	Seychelles Child Development Study Nutrition Cohort 2: 1265 mother-child pairs (children at age 20 months)	Total Hg in maternal hair at delivery and maternal weekly fish consumption	Mean maternal hair Hg: 3.92 (sd. 3.46) Maternal estimate of weekly fish meals: 8.52 (4.56)	No overall association of Hg with neurodevelopment, but evidence for possible interaction of Hg with fish oils for neurodevelopment: higher levels of Hg were negatively associated with psychomotor scores for children of mothers with higher ratio of n-6 to n-3 fatty acids; whereas higher Hg was positively associated with psychomotor development among children born to mothers with higher n-3 fatty acids
Seychelles (Davidson *et al*., 2008)	300 mothers and 229 children at ages 5, 9, 25 and 30 months	Number of fish meals per week of mother during pregnancy	Mean maternal hair MeHg: 5.9	Neurodevelopmental performance at 30 months decreased with increased MeHg, adjusted for nutritional factors
Tohoku, Japan (Tatsuta *et al*., 2014)	387 42-month old children	Cord blood total Hg levels	Median cord blood Hg: 0.01	No significant correlations between neurodevelopmental score and total mercury
New Zealand, North Island (Crump *et al*., 1998 re-analysis of Kjellström *et al*., 1986; Kjellström *et al*., 1989)	237 children ages 6–7 (paired with their mothers)	Average maternal hair Hg concentration during pregnancy	61 children with hair Hg > 6 ppm matched to lower-Hg-exposed children. Crump *et al.* use continuous hair Hg measures, but do not report average Hg value	Negative association of maternal hair Hg with academic attainment, language development, fine and gross motor coordination, and intelligence – after omitting one highly influential point from the analysis

IQR, inter-quartile range (25th and 75th percentiles of distribution).

### Immune toxicity of mercury

Data are limited regarding whether mercury from fish affects the immune system, although studies have been conducted in human populations and in toxicological experiments. In cross-sectional studies in Amazonian Brazil, elevated mercury exposures were associated with increased levels of auto-antibodies in gold miners highly exposed to elemental mercury but also possibly exposed to some methylmercury (Silva *et al*., 2004; Gardner *et al*., 2010a). A cross-sectional, nationally representative survey of adults in the USA, showed that hair and blood mercury (thought to largely reflect methylmercury exposures) but not urine mercury (thought to largely reflect inorganic exposures) were associated with having anti-nucleolar auto-antibodies (Somers *et al*., 2015). In *in vitro* toxicological experiments with sufficiently high doses of mercury (3.6 to 36 μM) to induce cell death within 24 h, exposure of human immune cells *in vitro* to methylmercury prevented B cell proliferation, and these suppressive effects were more severe if mercury exposure occurred prior to immune cell activation (Shenker *et al*., 1993). In T cells, proliferation was suppressed and apoptosis induced following mercury exposure *in vitro*, although these effects were examined in mixed culture systems (Shenker *et al*., 1992; Shenker *et al*., 1998). In mixed cultures of peripheral blood mononuclear cells stimulated with lipopolysaccharide, which stimulates macrophages, subcytotoxic concentrations of methylmercury increased production of pro-inflammatory cytokines TNF-α and IL-1β (Gardner *et al*., 2009, 2010b). Thus, stimulatory effects of methylmercury were observed at doses closer to the typical *in vivo* human exposure range, generally less than 200 nM (Mahaffey, 2004; Mahaffey *et al*., 2009), while higher doses were inhibitory. In the more environmentally relevant administered dose studies, effects were primarily observed when cells were stimulated, suggesting that immune activation state at least partially determines the sensitivity to toxic effects on the immune system. If mercury does affect inflammation, then inflammatory mechanisms could impact other organ systems including the cardiovascular system.

### Cardiovascular toxicity of mercury

Mercury's potential impacts on the cardiovascular system are a growing area of research (Roman *et al*., 2011). Mercury's relationship to fatal heart attacks was recently cited as the potentially most expensive and therefore the most important uncertainty in the cost-benefit analysis for economic benefit of mercury pollution reductions to the USA (Rice *et al*., 2010). Myocardial infarction and mortality risks from mercury have been evaluated in several recent studies. A cross-sectional survey in a nationally representative sample of South Koreans found a higher odds of previous myocardial infarction with higher levels of blood mercury (Kim *et al*., 2014). A case-control study of 1408 men found that toenail mercury was associated with higher odds of myocardial infarction after accounting for levels of the heart-protective fatty acid DHA (Guallar *et al*., 2002). In contrast, a pooled convenience sample drawn from the Health Professionals Follow-up Study and Nurses’ Health Study in the USA (6045 adults) found non-significant, but potentially protective associations between toenail mercury and risk of myocardial infarction, stroke and coronary heart disease (Mozaffarian *et al*., 2011). This result is acknowledged by the authors to likely reflect the cardio-protective benefits of fish oils, rather than being an accurate portrait of mercury's cardiovascular impact *per se*. A Swedish cohort also found lower risk of first myocardial infarction with higher erythrocyte mercury, even after controlling for a plasma biomarker of fish oils (Hallgren *et al*., 2001). In contrast, a large cohort study of 1871 elderly men in Finland found strong positive associations of hair mercury levels with acute coronary events, death, and with cause-specific mortality related to congestive heart failure and cardiovascular disease (Virtanen *et al*., 2005). Additional research is needed to clarify whether mercury is causally associated with fatal cardiovascular disease, and to tease apart the reasons for the apparently discrepant findings in the existing literature. It is likely that there are differing distributions of interacting and confounding variables (i.e. other dietary nutrients, or genetics) across these study populations. Data on geographic variation in joint distributions of nutrients and contaminants in seafood would provide important context for interpreting the human health literature.

## CONCENTRATIONS OF EPA + DHA

Variability up to 128-fold has been documented in EPA and DHA levels across fish species (Gladyshev *et al*., 2013). EPA and DHA contents in aquatic animals depend on both taxonomic and ecological factors (Makhutova *et al*., 2011; Gladyshev *et al*., 2012b; Lau *et al*., 2012); other factors such as an anthropogenic pollution (Gladyshev *et al*., 2012a) may also be important. Research on the possible impacts of fish health status on fish fatty acid content is limited, but suggests the relationships may be complex and organism-specific. In a recent experiment with cultured puffer fish (*Fugu rubripes*) with or without *Trichodina* infection, flat fish (*Paralichthys olivaceus*) with or without streptococcus infection, yellowtail (*Seriola quinqueradiata*) with or without jaundice, and amberjack (*Seriola purpurascens*) with or without *Photobacterium damselae* sp. *piscicida*, there was not a significant difference by fish disease status in the overall fish fatty acid composition in fish livers; however, liver DHA was significantly higher in the diseased fish than healthy fish for flat fish, yellowtail and amberjack (Tanaka *et al*., 2014). There is also growing interest in how oxidative stress in fish may affect fish lipids (Tanaka & Nakamura, 2012; Tanaka *et al*., 2014).

One objective for our review is to summarize data on EPA and DHA across fish populations. To identify EPA and DHA content of diverse marine fish species, including anadromous fish, we queried Web of Science, Core Collection on 2 October 2014 for ‘fatty acid AND content AND fish AND marine’ (Table 2). Unfortunately, most studies report EPA and DHA as per cent of total fatty acids, and do not provide quantitative information on contents of these PUFA in mass units per fish portion (Gladyshev *et al*., 2007, 2012b; Huynh & Kitts, 2009). In this manuscript, we review data from 10 studies reporting direct measurements of EPA and DHA contents in wild fish biomass obtained using internal standards in chromatography (using capillary columns) over two recent decades. These had slightly different methodologies. For small fish, less than 35 cm (e.g. sardine or capelin), the fish were analysed whole (Huynh & Kitts, 2009). Larger fish species (e.g. salmon) were sampled by dissecting muscle tissue (filets without skin), usually under dorsal fin (e.g. Gladyshev *et al*., 2006, 2007, 2012b; Huynh & Kitts, 2009; Kitson *et al*., 2009; Abd Aziz *et al*., 2013; Sahari *et al*., 2014). In some studies (Chuang *et al*., 2012) ventral muscles were sampled. In other studies both small and large fish were taken whole, e.g. ground and homogenized (Castro-Gonzalez *et al*., 2013). Some authors did not report the sampling in detail (Garcia-Moreno *et al*., 2013).

**Table 2.** Content of eicosapentaenoic (EPA) and docosahexaenoic (DHA) acids (mg g^−1^, wet weight) in various wild fish species, their types of habitat (H1: p, pelagic, bp, benthopelagic, d, demersal; H2: c, cold waters, t, temperate waters; w, warm waters) and size (cm). Orders and species are ranged by EPA + DHA content values.

Taxon	EPA	DHA	EPA + DHA	H1	H2	Size	Reference
*Order Clupeiformes*							
Sardine (*Sardinops sagax*)	6.60	19.00	25.60	p	t	30	Huynh & Kitts (2009)
Longtail shad (*Hilsa macrura*)	20.42	1.69	22.11	p	w	35	Abd Aziz *et al*. (2013)
Sardine (*Sardina pilchardus*)	8.50	8.37	16.87	p	t	25	Garcia-Moreno *et al*. (2013)
Round herring (*Etrumeus teres*)	12.34	4.33	16.67	p	t	25	Castro-Gonzalez *et al*. (2013)
Herring (*Clupea harengus*)	8.50	8.30	16.80	p	c	25	Huynh & Kitts (2009)
Rainbow sardine (*Dussumieria acuta*)	3.43	10.16	13.59	p	w	20	Sahari *et al*. (2014)
Fringescale sardinella (*Clupea fimbriata*)	2.11	2.25	4.36	p	w	25	Abd Aziz *et al*. (2013)
Dorab wolf-herring (*Chirocentrus dorab*)	0.24	0.54	0.78	p	w	100	Abd Aziz *et al*. (2013)
Shad (*Alosa alosa*)	0.12	0.43	0.55	p	t	45	Chuang *et al*. (2012)
*Order Salmoniformes*							
Atlantic salmon (*Salmo salar*)	6.20	5.80	12.00	bp	c	70	Kitson *et al*. (2009)
Pink salmon (*Oncorhynchus gorbuscha*)	1.70	3.30	5.00	d	c	50	Gladyshev *et al*. (2006)
Sockeye salmon (*Oncorhynchus nerka*)	0.70	1.90	2.60	p	c	50	Gladyshev *et al*., (2012a, b)
*Order Perciformes*							
Horse mackerel (*Trachurus mediterraneus*)	4.40	5.49	9.89	bp	t	25	Garcia-Moreno *et al*. (2013)
Spanish mackerel (*Scomberomorus commerson*)	1.60	7.72	9.32	p	w	90	Sahari *et al*. (2014)
Yellowstripe scad (*Selaroides leptolepis*)	0.97	7.82	8.79	d	w	15	Abd Aziz *et al*. (2013)
Horse mackerel (*Trachurus trachurus*)	1.64	5.86	7.50	bp	t	30	Chuang *et al*. (2012)
Axillary seabream (*Pagellus acarne*)	3.19	3.41	6.60	bp	t	25	Garcia-Moreno *et al*. (2013)
Oilfish (*Ruvettus pretiosus*)	1.13	5.33	6.46	d	t	150	Castro-Gonzalez *et al*. (2013)
Kawakawa (*Euthynnus affinis*)	0.93	5.51	6.44	p	w	60	Sahari *et al*. (2014)
Longjaw leatherjacket (*Oligoplites altus*)	1.05	5.02	6.07	bp	w	30	Castro-Gonzalez *et al*. (2013)
Japanese threadfin bream (*Nemipterus japonicus*)	2.59	2.93	5.52	d	w	25	Abd Aziz *et al*. (2013)
Broadbill swordfish (*Xiphias gladius*)	0.52	4.34	4.86	p	t	150	Castro-Gonzalez *et al*. (2013)
Atlantic tripletail (*Lobotes surinamensis*)	0.68	3.22	3.90	p	w	50	Castro-Gonzalez *et al*. (2013)
Black pomfret (*Parastromateus niger*)	0.73	2.77	3.50	p	w	30	Abd Aziz *et al*. (2013)
King mackerel (*Scomberomorus guttatus*)	0.45	3.02	3.47	p	w	45	Sahari *et al*. (2014)
Longtail tuna (*Thunnus tonggol*)	0.53	2.92	3.45	p	w	65	Sahari *et al*. (2014)
Parrot sand bass (*Paralabrax auroguttatus*)	0.98	2.21	3.19	d	w	50	Castro-Gonzalez *et al*. (2013)
Moonfish (*Trachinotus blochii*)	1.77	1.23	3.00	d	w	80	Abd Aziz *et al*. (2013)
Sixbar grouper (*Epinephelus fasciatus*)	1.01	1.98	2.99	d	w	25	Abd Aziz *et al*. (2013)
Silver pomfret (*Pampus argentus*)	1.16	1.48	2.64	p	w	30	Abd Aziz *et al*. (2013)
Malabar red snapper (*Lutjanus argentimeculatus*)	0.24	2.10	2.34	d	w		Abd Aziz *et al*. (2013)
Giant sea perch (*Lates calcarifer*)	1.39	0.95	2.34	d	w	80	Abd Aziz *et al*. (2013)
Sea bass (*Dicentrarchus labrax*)	0.52	1.75	2.27	d	t	50	Chuang *et al*. (2012)
Hardtail scad (*Megalapsis cordyla*)	0.19	1.96	2.15	p	w	35	Abd Aziz *et al*. (2013)
Bogue (*Boops boops*)	0.63	0.94	1.57	bp	t	20	Garcia-Moreno *et al*. (2013)
Fourfinger threadfin (*Eleutheronema tetradactylum*)	0.96	0.53	1.49	p	w	50	Abd Aziz *et al*. (2013)
Gray snapper (*Lutjanus griseus*)	0.45	1.03	1.48	d	w	40	Castro-Gonzalez *et al*. (2013)
Yellowfin tuna (*Thunnus albacares*)	0.13	1.30	1.43	p	t	150	Castro-Gonzalez *et al*. (2013)
Red mullet (*Mullus barbatus*)	0.48	0.94	1.42	d	t	15	Chuang *et al*. (2012)
Atlantic blue marlin (*Makaira nigricans*)	0.15	1.04	1.19	p	w	250	Castro-Gonzalez *et al*. (2013)
Indian threadfin (*Polynemus indicus*)	0.24	0.82	1.06	d	w	70	Abd Aziz *et al*. (2013)
Spanish mackerel (*Scromberomorus guttatus*)	0.28	0.7	0.98	p	w	45	Abd Aziz *et al*. (2013)
Indian mackerel (*Rastrelliger kanagurta*)	0.54	0.23	0.77	p	w	25	Abd Aziz *et al*. (2013)
American harvestfish (*Peprilus paru*)	0.08	0.57	0.65	p	w	18	Castro-Gonzalez *et al*. (2013)
Golden snapper (*Lutjanu sjohnii*)	0.07	0.19	0.26	d	w		Abd Aziz *et al*. (2013)
Brown meager (*Sciaena umbra*)	0.05	0.19	0.24	d	t	35	Chuang *et al*. (2012)
Bonito (*Sarda sarda*)	0.03	0.15	0.18	p	t	50	Chuang *et al*. (2012)
Spotted weakfish (*Cynoscion nebulosus*)	0.02	0.02	0.04	d	w	35	Castro-Gonzalez *et al*. (2013)
*Order Osmeriformes*							
Surf smelt (*Hypomesus pretiosus*)	3.60	5.70	9.30	p	t	15	Huynh & Kitts (2009)
Capelin (*Mallotus villosus*)	3.60	4.60	8.20	p	c	10	Huynh & Kitts (2009)
*Order Scorpaeniformes*							
Canary rock fish (*Sebastes pinniger*)	3.50	5.40	8.90	d	t	40	Huynh & Kitts (2009)
Spotted scorpionfish (*Scorpaena plumieri*)	0.22	2.28	2.50	d	w	25	Castro-Gonzalez *et al*. (2013)
Scorpion (*Scorpaena scrofa*)	0.29	1.40	1.69	d	t	30	Chuang *et al*. (2012)
*Order Gadiformes*							
Alaska pollock (*Theragra chalcogramma*)	1.00	2.40	3.40	d	c	60	Huynh & Kitts (2009)
Pacific hake (*Merluccius productus*)	0.90	1.50	2.40	d	t	60	Huynh & Kitts (2009)
Cod (*Gadus morhua*)	0.60	1.50	2.10	d	t	60	Gladyshev *et al*. (2007)
Whiting (*Gadus merlangus*)	0.08	0.48	0.56	d	t	35	Chuang *et al*. (2012)
*Order Pleuronectiformes*							
Rock sole (*Lepidopsetta bilineata*)	1.80	1.10	2.90	d	t	30	Gladyshev *et al*. (2007)
Largescale tonguesole (*Cynoglossus arel*)	0.08	1.13	1.21	d	w	30	Abd Aziz *et al*. (2013)
*Order Siluriformes*							
Gray eel-catfish (*Plotosus* spp.)	1.46	0.89	2.35	d	w		Abd Aziz *et al*. (2013)
*Order Mugiliformes*							
Mullet (*Mugil cephalus*)	0.46	0.08	0.54	p	t	50	Chuang *et al*. (2012)
*Order Beloniformes*							
Garfish (*Belone belone*)	0.01	0.15	0.16	p	t	70	Chuang *et al*. (2012)
*Order Myliobatiformes*							
Long-tailed butterfly ray (*Gymnura* spp.)	0.03	0.09	0.12	d	w		Abd Aziz *et al*. (2013)

The resulting data set includes 63 fish species across 11 orders (Table 2). Since PUFA contents in aquatic animals are known to depend on both phylogenetic and ecological factors (Makhutova *et al*., 2011; Gladyshev *et al*., 2012b; Lau *et al*., 2012), fish species were organized by their EPA and DHA values within taxonomic orders. Putative effects of ecological (habitat) factors were taken into account by dividing the fish species into pelagic, benthopelagic and demersal, as well as by category of water temperature of their habitat, i.e. cold-water, temperate and warm-water (tropical) species. Common size of the fish species was used as a proxy of their trophic level, although this is an imperfect surrogate.

**Fig. 1.**
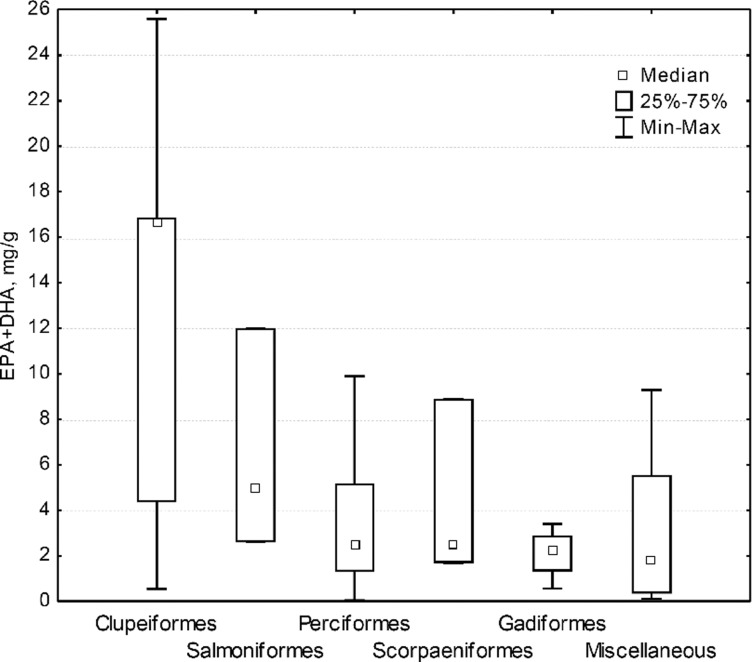
Contents of eicosapentaenoic acid (EPA) + docosahexaenoic acid (DHA) in fish orders: minimum, maximum and median values and quartiles. Number of species, N: order Clupeiformes, N = 9; order Salmoniformes, N = 3; order Perciformes, N = 36; order Scorpaeniformes, N = 3; order Gadiformes, N = 4; miscellaneous (orders Osmeriformes, Pleuronectiformes, Siluriformes, Mugiliformes, Beloniformes and Myliobatiformes), N = 8.

Values of EPA + DHA concentration in the 63 fish species varied from 25.60 mg g^−1^ (sardine *Sardinops sagax*, order Clupeiformes) to 0.04 mg g^−1^ (spotted weakfish *Cynoscion nebulosus*, order Perciformes) (Table 2). Clupeiformes had the highest median and maximum values of EPA + DHA contents, followed by Salmoniformes, while Perciformes, Scorpaeniformes and Gadiformes and miscellaneous had nearly similar median values (Figure 1). Nevertheless, ranges of values for EPA + DHA content of all orders overlapped in minimum values (Figure 1, Table 2). Thus, all orders, including Clupeiformes, have species with comparatively low content of EPA and DHA.

**Fig. 2.**
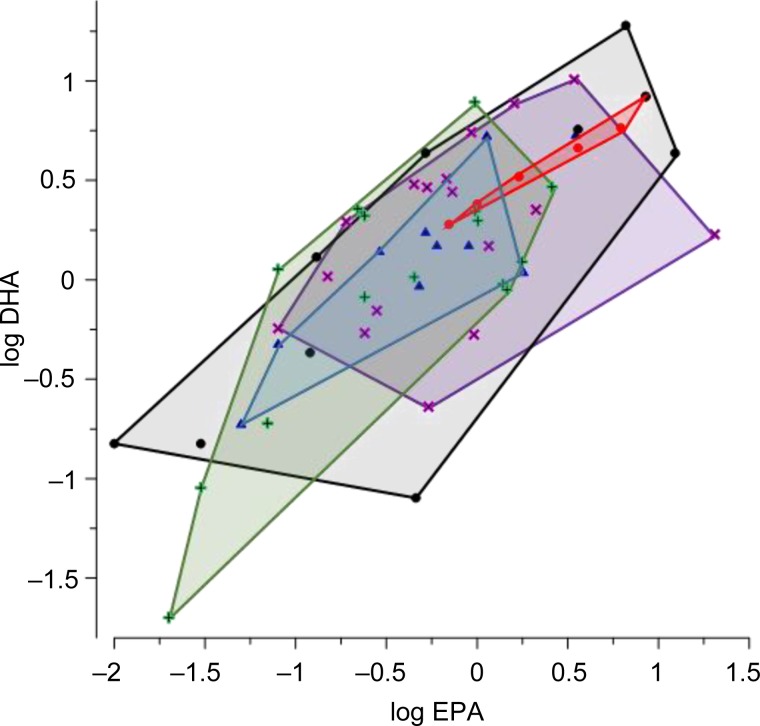
Areas of eicosapentaenoic acid (EPA) *vs* docosahexaenoic acid (DHA) A levels in fish species from diverse habitats: pelagic warm water species (number of species, N = 17, violet), pelagic temperate water species (N = 10, black), demersal warm water species (N = 15, green), demersal temperate water species (N = 10, blue) and cold water species (N = 6, red).

Interpretation of these results may be complicated by measurement error introduced by differing methods used for fish sampling and analysis, but some broad patterns in the data are interesting. Analysis of published EPA + DHA values found no statistically significant effect of type of habitat (pelagic, benthopelagic and demersal), or temperature of habitat, or their interaction on the PUFA content in fish. To visualize the results of ANOVA, a two-dimensional graph of the PUFA content in the groups of species was created (Figure 2). Since EPA + DHA contents in benthopelagic species overlapped completely with those of pelagic and demersal species, they were not included in the depicted groups. In addition, there were only six cold water species amongst pelagic, benthopelagic and demersal, which were joined in one group. The graph illustrates that EPA and DHA values of all the groups, pelagic temperate water, pelagic warm water, demersal temperate water, demersal warm water and cold water species, overlapped nearly completely.

This analysis of available data did not identify a strong predictor for EPA and DHA contents in fish. Temperature, for example, had limited impact: the contents of EPA + DHA in three pelagic planktivorous Clupeiformes with nearly identical sizes: sardine *Sardinops sagax* from temperate waters, shad *Hilsa macrura* from warm waters and herring *Clupea harengus* from cold waters were all similar (Table 2). Moths *et al*. (2013) analysed freshwater fish from the Great Lakes as well as 99 other species from freshwater and marine systems documented in seven other studies. As in this study, Moths *et al*. (2013) found that for marine systems, there was no relationship between latitude and omega-3 fatty acid composition of fish. However, in temperate climates, marine fish had higher omega-3/6 ratios than freshwater fish and for freshwater fish alone, there were higher omega-3 fatty acids in temperate fish as compared with tropical fish. While this study was based on relatively few datasets and many different species, it suggests some interesting patterns. For marine zooplankton, Kattner & Hagen (2009) did not find significant differences in latitudinal distribution of EPA and DHA levels. Since zooplankton are the main food of these three planktivorous fish species from different latitudes, Kattner & Hagen's (2009) findings for zooplankton are consistent with those for the planktivorous fish. Thus, more specific characteristics of diverse aquatic ecosystems, such as levels of primary production of PUFA and the efficiency of their transfer through trophic chains (Gladyshev *et al*., 2011), are likely to be contributing factors for EPA and DHA content of given fish species. In these large meta-analyses, many environmental and fish specific variables may obscure the potential effects of individual environmental factors such as temperature or trophic level, or pharmacokinetic compartment differences of lipids across fish tissues. More research directed to effects of fish phylogenetics, ecological niche, type of habitat, food quality and other possible determinants is needed to be able to predict EPA and DHA contents, particularly in marine fish.

Studies of fish from field sampling, particularly with heterogeneous methodology, are not conducive to investigating the mechanistic sources of difference between populations living in different environmental settings. In contrast to the analysis of metadata for fish fatty acids above, experimental laboratory studies suggest that fatty acid concentrations in plankton and fish may be influenced in part by the food and temperature environments to which they are exposed. Numerous studies have shown that EPA and PUFAs increase in cells grown at lower temperatures and saturated fatty acids decrease (Thompson *et al*., 1992; Jiang & Gao, 2004; Fuschino *et al*., 2011; Teoh *et al*., 2013). In addition, some fish either naturally occurring or cultured have higher concentrations of fatty acids when grown in colder temperatures. Fish need to adjust membrane fluidity for metabolic function in fluctuating temperatures (homeoviscous adaptation) and they do this by changing the concentrations and composition of individual fatty acids and sterols in cell membranes (Sinensky, 1974; Snyder *et al*., 2012). Several experimental studies show differences in fatty acid concentrations in fish exposed to different temperatures. Experiments with juvenile Atlantic salmon at two temperatures (14 and 19°C) found that *n*-3, *n*-5 and total fatty acids were higher in fish raised in colder water (Arts *et al*., 2012). Another study on cultured Atlantic salmon found that the temperature effect was dependent on the type of oil in their food; temperature effects were more pronounced in fish fed copepod oil diets than fish oil diets (Bogevik *et al*., 2011). Another study found the digestibility of the lipids in Atlantic salmon to increase with increasing rearing temperatures suggesting that while colder temperatures may favour higher fatty acid concentrations, they may be less digestible than at warmer temperatures (Huguet *et al*., 2015). Laurel *et al*. (2012) found that lower temperatures also favoured increases in unsaturated fatty acids in newly hatched Pacific cod larvae but relative amounts of essential fatty acids did not change with temperature. Similarly, *n*-3 and *n*-6 fatty acids decreased with increased temperatures in eggs and larvae of the marine fish, *Inimicus japonicas* (Wen *et al*., 2013). Thus, there are a range of experimental studies supporting the role of temperature and potentially diet determining fatty acid composition in aquatic plankton and fish. They suggest that colder temperatures result in higher amounts and differing quality of fatty acids. However, the disparity between patterns observed in experimental and field-based studies should be further investigated.

## VARIABILITY IN FISH MERCURY CONCENTRATIONS

One of the major challenges in managing human exposure to mercury from fish consumption is that fish mercury concentrations are highly variable. Numerous studies have measured broad differences in mercury content across different finfish and shellfish taxa (Sunderland, 2007; Karimi *et al*., 2012). A recent review estimated that mercury content within a given taxon can also be highly variable, ranging from 0.3–2.4 orders of magnitude, depending on the taxon (Karimi *et al*., 2012). This variability poses a challenge to estimating mercury exposure from seafood consumption, and makes it difficult to quantify the risk associated with consuming specific fish taxa.

Numerous studies have shown that body size, age, trophic level and food source of fish are related to concentrations of methylmercury and the per cent of total mercury that is methylmercury (Chen *et al*., 2009; Piraino & Taylor, 2009). Across species, body size can be more strongly correlated with mercury concentration than trophic level (Karimi *et al*., 2013). In general, larger fish across and within species have higher concentrations of methylmercury because larger fish eat higher trophic level prey and are older and have had a longer time to accumulate mercury (Cossa *et al*., 2012; Storelli and Barone, 2013). However, some studies have found that mercury concentration is more strongly correlated with age than length or weight (Braune, 1987; Burger & Gochfeld, 2011). For example, the size of Bluefin tuna is not related to mercury concentration (Burger & Gochfeld, 2011) and Atlantic herring in the Arctic show relationships at 3–5 years of age but a decrease at 1–2 years of age due to growth dilution (Braune, 1987). While there are clear positive relationships between total mercury and fish size and fish age, there is still variability in total mercury concentrations that is not explained by those two variables as well as the presence of interspecific and intraspecific variability (Tremain & Adams, 2012). Some of this unexplained variability likely comes from the food source and geographic range of the fish. Fish that have more pelagic than benthic food sources appear to bioaccumulate higher concentrations of mercury (Power *et al*., 2002; Chen *et al*., 2009; Karimi *et al*., 2013). Not surprisingly, fish that are exposed to higher water and sediment concentrations also have higher tissue concentrations of mercury (Lowery & Garrett, 2005; Chen *et al*., 2009; Gehrke *et al*., 2011; Taylor *et al*., 2012; Chen *et al*., 2014). However, levels of mercury may vary between similar species in a small geographic area and by tissue within a fish (Bank *et al*., 2007). A recent study also suggests increases in methylmercury bioaccumulation in fish experiencing warmer temperatures (Dijkstra *et al*., 2013). Overall, these studies show that fish size, age, trophic level, food source and geographic region each influence fish mercury content, with no strict rules for which of these factors explains the largest portion of mercury variability. While agencies such as the Food and Drug Administration (FDA) in the USA monitor mercury in marine fish consumed by humans, they do not report fish sizes or geographic location, both of which are extremely important when looking at mercury bioaccumulation.

## SELENIUM AND MERCURY CONCENTRATIONS IN FISH

There is a long-running interest in nutrient-toxicant interactions between mercury and selenium (Ganther *et al*., 1972). Although recent evidence suggests possible synergistic interactions between mercury and selenium for fish development (Penglase *et al*., 2014), the weight of evidence suggests antagonistic interactions in which selenium mediates mercury toxicokinetics (reviewed in Peterson *et al*., 2009). Selenomethionine increases mercury elimination in zebrafish (*Danio rerio*) (Yamashita *et al*., 2013; Amlund *et al*., 2015), shrimp (Bjerregaard & Christensen, 2012) and goldfish (*Carassius auratus*) (Bjerregaard *et al*., 2012); selenite, and seleno-cysteine also increased mercury elimination in goldfish and shrimp. In humans, dietary organic selenium can increase mercury elimination (Li *et al*., 2012). Ralston and colleagues report that selenium not only ameliorates the toxic effects of methylmercury by sequestering methylmercury and reducing its bioavailability to organisms, but mercury and selenium may also have physiologically important interactions mediated by other mechanisms (Ralston *et al.*, 2007; Ralston & Raymond, 2010). Based on rat data, Ralston (2008) suggests that where the selenium to mercury molar ratio exceeds 1:1, there is adequate selenium to counter mercury toxicity. However, this has not been clearly demonstrated in humans. In recent trout (*Salmo trutta*) studies in a Norwegian lake, the selenium to mercury molar ratio was a better predictor of trout metallothionein levels than was either selenium or mercury (Sørmo *et al*., 2011). However, human studies and clinical trials for selenium demonstrate mixed and inconclusive results for cardiovascular effects of methylmercury and selenium (Mozaffarian, 2009). It has been suggested that mercury cardiovascular toxicity may be modified by selenium intake (Cooper *et al*., 2007; Khan & Wang, 2009; Mozaffarian, 2009). This might arise through selenium impacts on mercury kinetics (Huang *et al*., 2013) or through impacts on oxidative stress mediators of mercury toxicity (Kaneko & Ralston, 2007; Ralston *et al*., 2007; Farina *et al*., 2011; Alkazemi *et al*., 2013; Drescher *et al*., 2014), although evidence for the oxidative stress mediation hypotheses is ambiguous (Belanger *et al*., 2008). Selenium-mercury interactions may also be relevant for neurodevelopmental outcomes (Choi *et al*., 2007).

In recent years due to the interest in selenium to mercury molar ratios, a number of studies have assessed mercury and selenium concentrations and the selenium to mercury molar ratios for a variety of fish species from field samples as well as fish purchased from supermarkets (Burger *et al*., 2005, 2013; Burger & Gochfeld, 2011, 2012; Gochfeld *et al*., 2012; Karimi *et al*., 2013, 2014). The relationship between body size and selenium to mercury molar ratios vary with species, tissues and geographic location. Selenium to mercury molar ratios decreased with size of fish for yellowfin tuna and windowpane flounder in Delaware Bay and a wide variety of species in the Aleutians (Burger & Gochfeld, 2011, 2012). Some individuals of most of the 15 species studied in the Aleutians had ratios less than 1.0, where older, larger, higher trophic level fish had the lowest ratios. This was the result of mercury concentrations increasing with fish size but selenium concentrations not increasing with size. While selenium to mercury molar ratios were negatively correlated with fish length for bluefish, the ratios were lower for white muscle tissue, the portion of the fish that humans consume. In a study of 19 species off the coast of New Jersey (USA), (Burger & Gochfeld, 2011) mercury and selenium were positively related for five species and negatively related for two species, and across all species, selenium had no consistent relationship with length. However, for most species tested across all of these studies, the ratios were greater than 1.0, although 20% of the striped bass caught by trawling off the New Jersey coast had molar ratios of less than 1.0 (Gochfeld *et al*., 2012).

In general, studies of selenium to mercury molar ratios have found that mercury concentrations were positively related to fish length and trophic level but selenium concentrations were not, and selenium to mercury molar ratios are more strongly related to mercury content than selenium content (Karimi *et al*., 2013). This reflects the fact that mercury more strongly accumulates in the body, and biomagnifies through the food chain compared with selenium (Karimi *et al*., 2013). These findings are consistent with lower efflux (loss) rates of methylmercury than selenium, because lower efflux rates lead to greater bioaccumulation over time as body size increases (Karimi *et al*., 2010). However, bivalves (e.g. clams, mussels and oysters) are known to be relatively efficient selenium accumulators (Stewart *et al*., 2004; Presser & Luoma, 2010), and have higher selenium concentrations than finfish (Karimi *et al*., 2013). It also appears that the mean selenium to mercury molar ratio declines with mean size of fish species and with individual fish size within a species. Both suggest that larger, predatory fish as well as the largest individuals of many species have lower selenium to mercury molar ratios and may not provide selenium protection against mercury toxicity for human seafood consumers (although selenium may be available in their diet from other sources). Moreover, smaller fish of a given species may provide greater protective benefits suggesting that those age classes that reside in more estuarine and coastal environments may present lower human health hazards (Burger *et al*., 2013). However, the variability of selenium to mercury molar ratios found within and across species makes it difficult to use this ratio in risk assessment, risk management and risk communication at the present time. Most governmental organizations that develop fish consumption advisories do not have the data on both mercury and selenium levels in individual fish which are necessary to determine the selenium to mercury molar ratio variation within and across species. It is difficult for risk assessors to develop advisories that are protective without an estimate of this variability.

## FISH THAT OPTIMIZE POTENTIAL BENEFITS VS RISKS

**Fig. 3.**
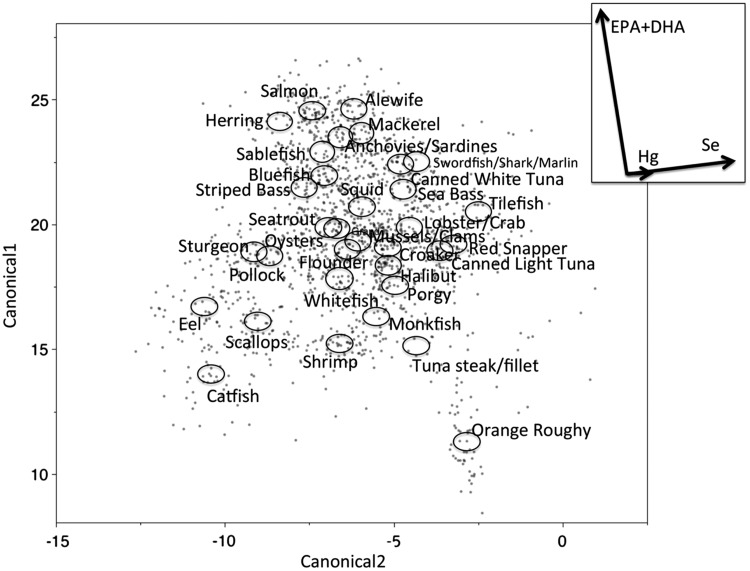
Canonical discriminant analyses testing for differences in mercury-nutrient signatures among seafood items (from Karimi *et al*., 2014, reprinted with permission). Circles indicate 95% confidence limits for means of each seafood group and indicate the degree of difference among groups. Mercury and nutrient vectors (inset) represent the underlying structure of the axes. The position of circles relative to the direction of vectors indicates correlations between seafood groups and the concentration gradient of mercury or nutrients. Vector length indicates the overall contribution of mercury or nutrients in discriminating among seafood groups. Vector direction indicates the correlation of mercury or nutrient with each axis (vectors parallel to an axis are highly correlated with that axis). Angles between vectors represent correlations among mercury and nutrient concentrations. EPA, eicosapentaenoic acid; DHA, docosahexaenoic acid; Hg, mercury; Se, selenium.

Recent research is beginning to address the need to quantify the overall nutritional and toxicological value of different types of fish and shellfish based on concentrations of multiple nutrients and contaminants in edible tissues. A recent study found unique, relative concentrations of mercury, omega-3 fatty acids, and selenium, or mercury-nutrient signatures, across seafood taxa (Figure 3, Karimi *et al*., 2014). Specifically, salmon and forage fish (herring, anchovies and sardines) are high in EPA and DHA compared with other seafood (Figure 3). In contrast, predatory fish have higher mercury concentrations than lower trophic level fish but nutrient concentrations do not appear to differ as strongly by trophic level. Karimi *et al*. (2014) found that these distinct mercury–nutrient signatures were reflected in the blood of seafood consumers based on their consumption habits. Most notably, consumers with a salmon-dominated diet had a high percentage of omega-3 fatty acids in their blood compared with other seafood consumers. Consumers who tended to eat top-predator fish had higher mercury, but similar nutrient concentrations in blood compared with consumers of lower trophic level seafood. These results suggest that consuming lower trophic level seafood can minimize the risk of mercury exposure without reducing the benefits of nutrient intake, and more broadly, demonstrate the value of examining nutrient and mercury exposure patterns simultaneously. Such research efforts are valuable in summarizing the largest signals among otherwise complex patterns of multiple nutrients and contaminants, but there is a need for a deeper understanding of these multivariate patterns at higher levels of taxonomic resolution. In some cases, the seafood categories used in this study include multiple species that share market names in order to compare mercury–nutrient signatures between edible seafood and seafood consumers. For example, salmon includes Atlantic salmon and multiple species of Pacific salmon, and tuna steak includes bigeye and yellowfin tuna (Karimi *et al*., 2014). Future studies that examine the composition of individual fish of the same species would complement these broader analyses by examining nutrient-contaminant patterns at greater taxonomic resolution, and in relation to ecological and environmental factors. In addition, better information on the taxonomic identity of market fish and shellfish would improve estimates of co-exposure to nutrients and contaminants in seafood consumers.

Advice describing both the types and amounts of seafood consumption, while complex, is necessary to better manage risks and benefits of seafood consumption (Oken *et al*., 2005; Gerber *et al*., 2012). Seafood risk communication also requires risks and benefits to be considered together for appropriate context (Kuntz *et al*., 2010; Turyk *et al*., 2012; Laird *et al*., 2013). Many fish advisories consider multiple chemical contaminants but provide minimal discussion of fish nutrients, focused on omega-3 fatty acids (Scherer *et al*., 2008). Compared with mercury concentrations, there are fewer studies quantifying fatty acids and selenium in seafood (Karimi *et al*., 2014). Therefore, to inform risk assessment more research is needed quantifying the risks and benefits associated with specific seafood consumption habits, such as considering the recommended daily consumption of seafood nutrients relative to reference doses (i.e. hazard quotients) of seafood contaminants (i.e. Gladyshev *et al*., 2009).

To conduct appropriate human health risk assessment for contaminants such as mercury requires an understanding of how mercury, fish oils and selenium co-exposures affect the human body. This work can be informed by studies from marine biology and fisheries science, coupled with epidemiological biomonitoring, anthropological and food science investigations into the role of culinary preparation and gut processing on mercury and nutrient bioavailability (Laird *et al*., 2009; Moses *et al*., 2009a, b; Costa *et al*., 2013). Acknowledging the concerns about contaminant exposure from seafood and its health benefits, the Joint FAO/WHO Expert Consultation on the Risks and Benefits of Fish Consumption (2010) recommended that government entities ‘Develop, maintain and improve existing databases on specific nutrients and contaminants, particularly methylmercury and dioxins, in fish consumed in their region’ and ‘Develop and evaluate risk management and communication strategies that both minimize risks and maximize benefits from eating fish’ (FAO/WHO, 2010, p. 33). Nevertheless, their general conclusions acknowledge fish as an important food source with clear benefits for reducing heart disease mortality and supporting optimal neurodevelopment in children.

## CONCLUSIONS

Our current ability to properly estimate the risks and benefits to humans of seafood consumption are hampered by the common approaches of separately studying either contaminants or nutrients in fish. To date there are few studies in which fish tissue concentrations have been measured for both contaminants and nutrients across a range of species and geographic regions, even for the restricted set of chemicals considered in this review. There is tremendous variability between and within fish species in their mercury, EPA and DHA concentrations, leading to different versions of the ‘fish intake’ exposure across participants in epidemiological studies (Greenland & Robins, 2009), complicating the interpretation of studies on seafood health implications. Better characterizing the extent of interspecies and intraspecies variation of chemicals in fish may help inform future human exposure studies by allowing for more explicit accounting of measurement error (Spiegelman *et al*., 1997; Murad & Freedman, 2007; Guo *et al*., 2012; Pollack *et al*., 2013). Furthermore, statistical methods are improving for epidemiological studies to incorporate source (i.e. seafood) contaminant levels, intake frequencies, toxicokinetic processes and biomarkers for an integrated exposure assessment (Conti *et al*., 2003; Bartell & Johnson, 2011; Tan *et al*., 2012; Shin *et al*., 2014); or to consider complex interactions between multiple seafood contaminants (Lynch *et al*., 2011) Thus, additional research on the joint distribution of multiple chemicals in marine foods has potential to contribute directly to future epidemiological investigations. Bringing multiple stakeholders (i.e. fish consumers and marine scientists) together in a trans-disciplinary conversation with health scientists can also help target the science to relevant questions and improve on knowledge translation (Boote *et al*., 2002; Burger *et al*., 2013). Future assessments of the risks and benefits of fish consumption will require more detailed understanding of exposures to both fish contaminants and nutrients as well as the environmental and ecological drivers that control their chemical transformations, and flow through marine food webs. The processes affecting composition of marine fish may be altered by climate change impacts including but not limited to ocean warming and ocean acidification (Edwards & Richardson, 2004; Halpern *et al*., 2008; Kroeker *et al*., 2012); fishing (Micheli *et al*., 2014); emerging joint exposures such as pharmaceuticals and personal care products potentially changing xenobiotic kinetics for some other compounds (Smital *et al*., 2004; Epel *et al*., 2008; Bosnjak *et al*., 2009); and future changes in contaminant sources and inputs (UNEP, 2013). Together, these changes indicate a need for continued research on fish nutrients and contaminants in marine and medical science, as well as ongoing communication between these disciplines.
